# An Objective Fluctuation Score for Parkinson's Disease

**DOI:** 10.1371/journal.pone.0124522

**Published:** 2015-04-30

**Authors:** Malcolm K. Horne, Sarah McGregor, Filip Bergquist

**Affiliations:** 1 Florey Institute for Neuroscience and Mental Health, University of Melbourne, Parkville, Victoria, Australia; 2 Centre for Clinical Neurosciences and Neurological Research, St Vincent’s Hospital Melbourne, Fitzroy, Victoria, Australia; 3 Department of Pharmacology, Institute of Neuroscience and Physiology, Sahlgrenska Academy, University of Gothenburg, Gothenburg, Sweden; Oslo University Hospital, NORWAY

## Abstract

**Introduction:**

Establishing the presence and severity of fluctuations is important in managing Parkinson’s Disease yet there is no reliable, objective means of doing this. In this study we have evaluated a Fluctuation Score derived from variations in dyskinesia and bradykinesia scores produced by an accelerometry based system.

**Methods:**

The Fluctuation Score was produced by summing the interquartile range of bradykinesia scores and dyskinesia scores produced every 2 minutes between 0900-1800 for at least 6 days by the accelerometry based system and expressing it as an algorithm.

**Results:**

This Score could distinguish between fluctuating and non-fluctuating patients with high sensitivity and selectivity and was significant lower following activation of deep brain stimulators. The scores following deep brain stimulation lay in a band just above the score separating fluctuators from non-fluctuators, suggesting a range representing adequate motor control. When compared with control subjects the score of newly diagnosed patients show a loss of fluctuation with onset of PD. The score was calculated in subjects whose duration of disease was known and this showed that newly diagnosed patients soon develop higher scores which either fall under or within the range representing adequate motor control or instead go on to develop more severe fluctuations.

**Conclusion:**

The Fluctuation Score described here promises to be a useful tool for identifying patients whose fluctuations are progressing and may require therapeutic changes. It also shows promise as a useful research tool. Further studies are required to more accurately identify therapeutic targets and ranges.

## Introduction

While identifying and managing motor fluctuations is central to managing the motor symptoms of Parkinson’s Disease (PD), there is no simple or objective measure of fluctuations. As the duration of efficacy of levodopa progressively shortens[1–4], fluctuations manifest as dyskinesias and re-emergence of tremor or bradykinesia prior to the next dose[1–8]. Approximately 40% of patients develop fluctuations and dyskinesia after 4–6 years of treatment and 70% after long-term treatment (>9 years). Recently we described an accelerometry based system for automated assessment of dyskinesia and bradykinesia[9]. This system has two algorithms that every two minutes, provides a score of the likelihood of movements being either dyskinetic or bradykinetic (dyskinesia score (DKS) and bradykinesia score (BKS) respectively. As a result, even the movement of subjects without PD (non-PD subjects) can have BKS and DKS and these scores will have a mean and distribution. A patient with PD is assessed by comparing the extent to which the distribution of their scores deviated from those of non-PD subjects. Fluctuations, by their name, imply that a measure of bradykinesia and dyskinesia, such as the BKS and DKS, would change over the course of the dose: in other words there would be greater variation in these scores than in a person whose dyskinesia and bradykinesia were relative constant over the course of the dose. Thus, a measure of this variation might reflect the extent of fluctuations.

Variations in the motor state can be caused by dyskinesia as well as from “wearing-off” without dyskinesia. Thus an examination of variation in both bradykinesia and dyskinesia scores is warranted. The Interquartile Range (IQR) is well accepted as a measure of variation of a non-parametric distribution such as the BKS and DKS distributions. In this study we examine the validity of combining variations in the BKS and DKS to produce a Fluctuation Score (FS) for assessing fluctuations in PD.

We provide evidence that the FS can distinguish between fluctuators and non fluctuators and measure the change that occurs following DBS. We conclude that the FS, as defined here, is a promising tool for studying fluctuations in PD. We propose that the FS may be useful for detecting people who are candidates for advanced therapy and for optimising oral therapies.

## Methods

**Fig 1:**
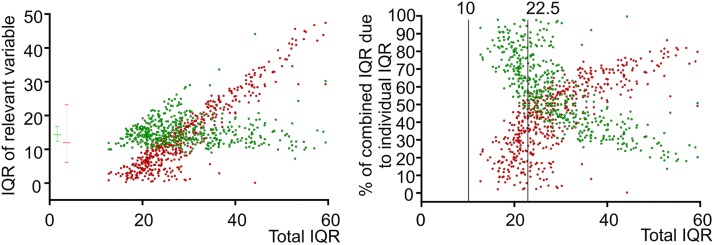
The interquartile range of bradykinesia and dyskinesia scores. A. The BKS_IQR_ (green dots) and DKS_IQR_ (green dots) for each patient was plotted against the IQR_C_. The error bars adjacent to the Y axis represent the median and interquartile range of the BKS_IQR_ (green lines) and DKS_IQR_ (red lines) shown in this figure. B. The BKS_IQR_ (green dots) and DKS_IQR_ (green dots) for each patient was expressed as a percentage of the IQR_C_ and plotted against the IQR_C_. Note that there no IQR_C_ values are less than 10. Note that the vertical line at IQR_C_ = 22.5, where the BKS_IQR_ contributes to about 60% to the IQR_C_, corresponds to the point that separates fluctuators and non fluctuators in Fig 2, showing that both BKS_IQR_ and DKS_IQR_ contribute significantly to the IQR_C_ at this point.

All assessments, including the use of the Parkinson’s KinetiGraph (PKG) and clinical rating scales were performed as part of routine care or as part of disparate research studies which received approval from the St Vincent’s Hospital Human Research & Ethics Committee. This committee provided approval Number was QA 072–14 to review the medical records of patients whose data was used in this paper was given as a quality assurance study. Thus the committee waived the need for written informed consent from the participants consent and records and information was made anonymous and de-identified prior to analyses. In total data from 527 records of people with idiopathic levodopa responsive PD and 38 control subjects were examined. Of these, 395 PD subjects were only used for the data described in Fig 1 and were selected because they had PD and had undergone a PKG recording. The remaining 132 were also used for the data in Fig 1 but were also used in all the subsequent studies and the basis for their selection are described in the following paragraphs. Fifty-two percent of these 132 patients were male and the average age of 132 subjects was 66 years (SD ±7.6 years), with on average, 7.1 years duration of disease.

### Fluctuators and non-fluctuators

**Fig 2:**
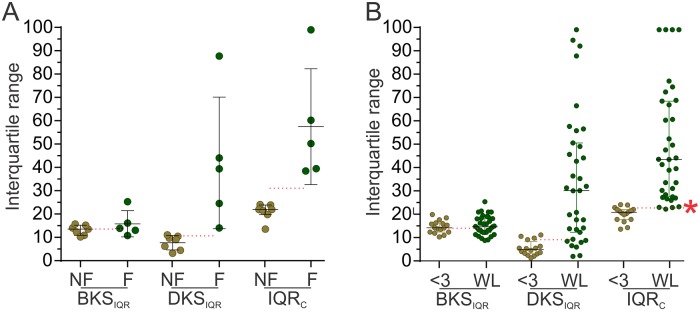
The interquartile range of bradykinesia and dyskinesia scores in fluctuators and non fluctuators. A. The BKS_IQR_, DKS_IQR_ and IQR_C_ of fluctuators (F and olive colour) and non fluctuating (NF and olive colour) patients were plotted. While the two populations can be separated using either DKS_IQR_ or IQR_C_, the separation is more distinct for the IQR_C_. In each plot, the horizontal red dotted lines show values from receiver operator curves that resulted in the highest selectivity and sensitivity for separating the two populations. In the case of the IQR_C_ plots the red dotted line corresponds to an IQR_C_ of 31.4 which separates the two groups with greater sensitivity and specificity than either the BKS_IQR_ or DKS_IQR_. Note that all IQR_C_ >100 are shown as = 100. Error bars show median and interquartile range. B. This is a plot of the BKS_IQR_, DKS_IQR_ and IQR_C_ of patients with PD for less than 3 years (<3 and olive colour) and of patients on the waiting list for DBS (WL and olive colour). In each plot, the horizontal red dotted lines show values from receiver operator curves that resulted in the highest selectivity and sensitivity for separating the two populations. In the case of the IQR_C_ plots the dotted line corresponds to an IQR_C_ of 22.5 which separates the two groups with greater sensitivity and specificity than either the BKS_IQR_ or DKS_IQR_. This is marked by an asterisk and is the value that becomes the FT and corresponds to an FS of 7.7 when the fluctuation formula is applied). Note that all IQR_C_ >100 are shown as = 100. Error bars show median and interquartile range.

Sixty four subjects were used in the study described in Fig 2. Patients were considered “fluctuators” if their treating Movement Disorder Specialist had observed or obtained a history of “wearing-off” or of dyskinesia. Non-fluctuators were patients in whom this history was not present. These studies are described more fully in the results, but the subjects in the pilot study, whose results are shown in Fig 2A, were known by one of us (FB). In the case of the subjects whose results are shown in Fig 2B, 36 patients on the waiting list for DBS were selected because their presence on the waiting list indicates that their treating clinicians considered them to be fluctuators. Because early fluctuators can be readily overlooked, 16 patients with disease of less than 3 years (some of whom were untreated) were used as “non-fluctuators”.

### DBS study

This was an observational study of 15 patients who were selected for surgery by their treating neurologist as part of routine care. The clinical rating scales (modified Abnormal Involuntary Movement Score (AIMS) and Unified Parkinson’s rating Scale part III (UPDRSIII)) and the collection of PKG data were performed at the centre undertaking the surgery. Based on clinical rating scales there was significant dyskinesia (modified active AIMS >7) in 50% of cases and the median UPDRS III was 23 (with or without tremor scores removed). Post-surgery rating was performed 6 months after commencement of stimulation.

### Approach to presentation of data

**Fig 3:**
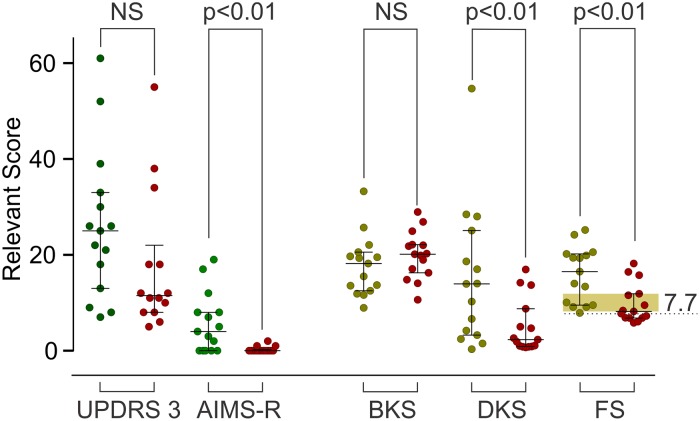
The Fluctuation Score before and after Insertion of Deep Brain Stimulators. Graphs showing the median and interquartile range of rating scores and PKG measures before and after DBS in 15 PD patients. The Y axis is the relevant value of the scale or PKG measure. The FS threshold for transition from non fluctuators to fluctuators (see asterisk Fig 2B) is indicated by a dotted line marked 7.7. The shaded orange region is the area between the median and 75^th^ percentile of FS (= 12.8) after DBS (See text for discussion). Note that the median FS after DBS (8.2) is very close to the FT: the value that separates fluctuators from non fluctuators in Fig 2 (FS = 7.7) Error bars show median and interquartile range. P values are obtained using Mann Whitney.

**Fig 4:**
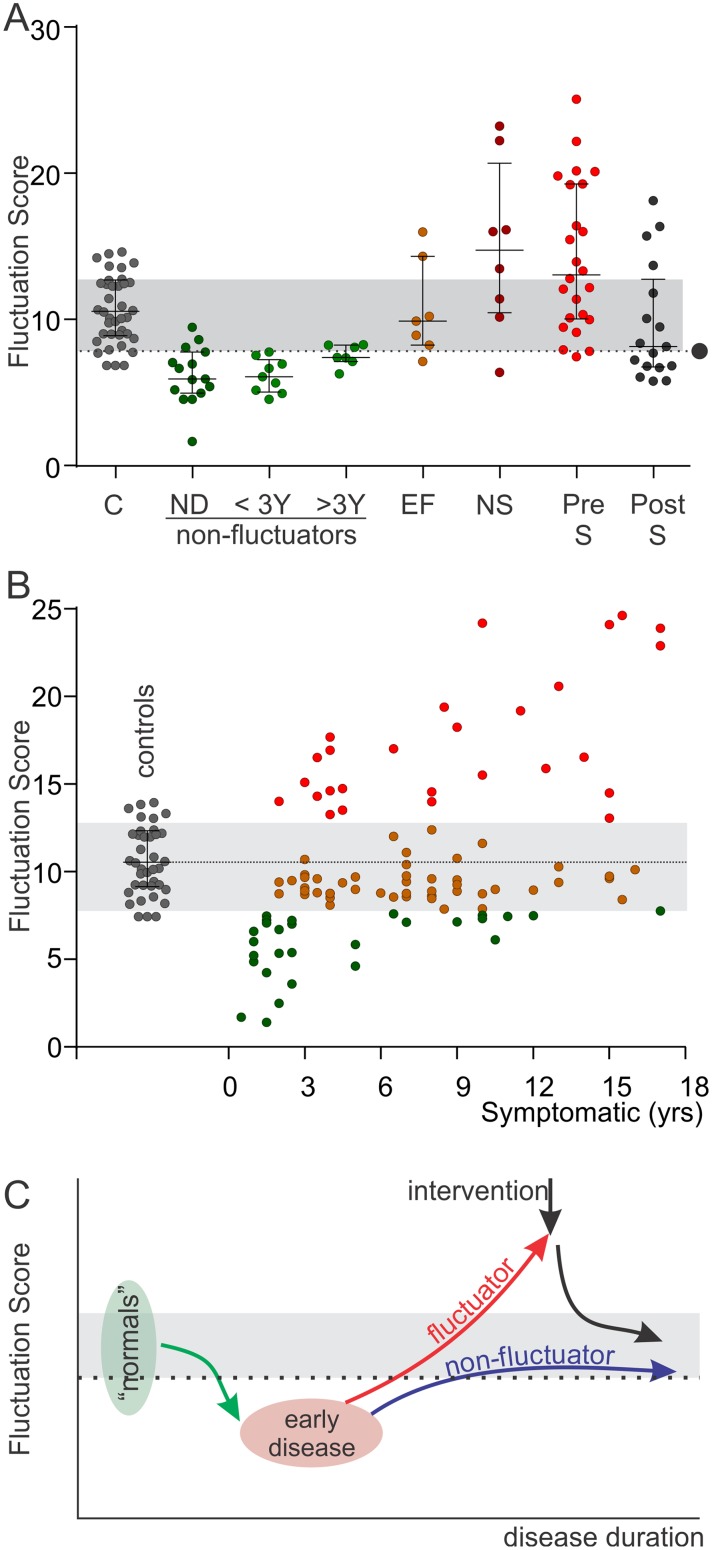
The change in Fluctuation Score with disease progression. A: The Fluctuation Score of subjects described by the following captions were plotted. C: Control subjects (grey dots). ND: people with PD who were newly diagnosed (green dots). <3Y: non fluctuators with disease duration less than 3 years, (green dots). >3Y: non fluctuators with disease duration more than 3 years (>3Y) (green dots), EF: “early” fluctuators (EF) meaning modest non troublesome fluctuations (brown dots), NS: fluctuators but not suitable (NS) for DBS (dark red dots), PRE-S: on the waiting list for DBS (crimson red dots). POST-S: Post DBS (black dots). The dotted line with a black circle is the FS scores that separates fluctuators and non-fluctuators (the FT) and the grey shaded region is the region between the FS scores for the median and 75^th^ percentile of Post DBS subjects (as in Fig 3). Note that this coincides with the interquartile rage of controls. Note the reduction of the FS score which is described in the results section and in the Discussion. B): The FS score of 177 subjects plotted against duration since first symptoms. Subjects whose FS is blow the FT are shown as green dots. Subjects with fluctuations within the RCMS are shown as tan dots and subjects with FS above the RCMS as red dots. The FS of Control subjects (from Fig 4A) are included (grey dots). Note that the black horizontal dotted line is a continuation of the median of controls. The FS scores within or above the RCMS became frequent after 3 years. Some subjects who had disease for many years had FS scores below the median of controls (dotted line) or even below the FT (see Discussion). C: This is a cartoon depicting the possible changes in FS over the course of PD and is based on Fig A and B above. The orange horizontal band represents the range in FS produced by an intervention such as DBS with the upper limit being the upper level of an acceptable response and the lower limit being FT (shown by the dotted line). Our proposal is that as non-PD subjects have an FS in this band and newly diagnosed PD are at a point below the cross-over point, then PD must initially produce a decline in FS until a diagnosis is made. With treatment and time, many subjects will have a progressive increase in their FS, eventually crossing the cross-over point and finally becoming a frank fluctuator and suitable for an intervention.

The approach to establishing an FS score is described in the results. Briefly, we first examined the variation in the distribution of BKS and DKS (as measured by their interquartile ranges) in 527 subjects (Fig 1). This led us to suggest that the sum of the interquartile ranges of the BKS and DKS might be useful as a measure of fluctuation. This was examined first in a small pilot cohort of fluctuators and non fluctuators (Fig 2B) and then in a larger cohort of people with early PD (non fluctuators) and those on the waiting list for DBS (fluctuators). This led us to conclude that sum of the interquartile ranges of the BKS and DKS could be used to distinguish between fluctuators and non fluctuators. This value was then express as a logarithm and called the Fluctuation Score (FS). This FS was shown to be significantly different before and after DBS and that the response to DBS could be used to define a range representing control of motor symptoms (Fig 3). We then examined how the FS changed over the progression of disease (Fig 4A and 4B).

### The natural history of the FS

The patients described in Fig 4B were 177 subjects selected from patient records because they had worn the PKG logger for more at least 6 days, whose duration of disease was known and who had also been assessed using AIMS. The patients described in Fig 4A are the patients described in the early figures but presented together and also included 38 subjects without PD (see below).

### Controls Subjects

Control subjects were aged 45–85 years and were required to wear the PKG for at least 6 days and to use the PKG under the same conditions as the PKG was used by people with PD.

### Recording Protocol

The Parkinson Kinetigraph data logger[9] (PKG, Global Kinetics Australia) was used for recording bradykinesia and dyskinesia[9]. This logger is worn on the wrist most severely affected by Parkinsonism and contains an accelerometer and memory sufficient for >10 days of continuous recording. In this study the PKG was worn for at least 6 days. When recording was completed, data was downloaded and analysed by proprietary algorithms that calculate a bradykinesia score (BKS) and a dyskinesia score (DKS)[9]. The algorithm produces BKS and DKS every two minutes and the median of these scores from the period between 0900–1800 from all recording days correlates with the UPDRSIII score (in the case of BKS) and the Abnormal Involuntary Movement Score (AIMS, in the case of the DKS)[9]. Tremor does not affect the BKS and in only rare cases does it affect the DKS.

### Clinical Rating Scales

A modified Abnormal Involuntary Movement Score (AIMS) and Unified Parkinson’s rating Scale part III (UPDRSIII) were performed on all subjects at the time they presented to the clinic, and the PKG was usually fitted at that time. Subjects had consumed their normal medications without specific regard to the time in their dosing cycle.

### Approach to presentation of data

The approach to establishing an FS score is described in the results. Briefly, we first examined the variation in the distribution of BKS and DKS (as measured by their interquartile ranges) in 527 subjects (Fig 1). This led us to suggest that the sum of the interquartile ranges of the BKS and DKS might be useful as a measure of fluctuation. This was examined first in a small pilot cohort of fluctuators and non fluctuators (Fig 2B) and then in a larger cohort of people with early PD (non fluctuators) and those on the waiting list for DBS (fluctuators). This led us to conclude that sum of the interquartile ranges of the BKS and DKS could be used to distinguish between fluctuators and non fluctuators. This value was then express as a logarithm and called the Fluctuation Score (FS). This FS was shown to be significantly different before and after DBS and that the response to DBS could be used to define a range representing control of motor symptoms (Fig 3). We then examined how the FS changed over the progression of disease (Fig 4A and 4B).

## Results

The prediction that the size of the Interquartile Range (IQR) of either or both the BKS and DKS provided by the PKG that will reflect the extent of fluctuations was examined. The IQR of the BKS and DKS (BKS_IQR_ and DKS_IQR_) were extracted from all 527 PKG recordings on the St Vincent’s Clinic data base and the sum of these (combined IQR (IQR_C_)) for each patient was calculated. By plotting the individual BKS_IQR_ and DKS_IQR_ (Fig 1A) and the individual BKS_IQR_ and DKS_IQR_ as a percentage of the IQR_C_ (Fig 1B), it was apparent that the BKS_IQR_ is greater than the DKS_IQR_ when the IQR_C_ is <~22.5. As the IQRs are measures of variation in the score, it implies that the DKS_IQR_ dominates when IQR_C_ is large, whereas variability of the BKS (the BKS_IQR_) is more significant when IQR_C_ is lower.

### Can the IQR_C_ distinguish fluctuators from non fluctuators

The validity of these IQR measures was first tested against a small sample of subjects with (n = 5) and without (n = 7) fluctuations (Fig 2A and see Methods for definitions). While these two populations were separated by the DKS_IQR_ alone, the separation was greater with the IQR_C_, although the area under the Receiver Operator Curve (ROC) for both IQR_C_ and DKS_IQR_ was 1.0.

The IQR_C_ was further tested in a second group of fluctuators (n = 36) who were on the waiting list for insertion of Deep Brain Stimulators (DBS) and a group of non fluctuators (n = 16), whose duration of PD was three years or less (Fig 2B). The median AIMS for those on the waiting list was 7 (range 1–23) and there was no dyskinesia (measured by AIMS) in the non fluctuators. The median IQR_C_ of the non fluctuators was 20.8 (interquartile range: 18–22) and 43.4.0 (interquartile range:27–69) for those on the waiting list. There was a significant difference between these two populations (p<0.0001: Mann Whitney). The area under the ROC for IQR_C_ was 0.98 and provided a sensitivity of 97.1% and selectivity of 87.5% at an IQR_C_ of 22.5. In comparison, the sensitivity and selectivity of the DKS_IQR_ was 80% and 81% and examination of Fig 2 shows the degree of overlap. Thus, contributions in the variation of both the BKS and DKS are required to establish a cut-off point between fluctuators and non fluctuators. We will refer to this cut-off point as the Fluctuation Threshold (FT). It is of interest that in Fig 1, the cut-off point between fluctuators and non fluctuators (the line marked by 22.5) is at a point where the BKS_IQR_ contributes more than 50% of the IQR_C_.

### Creating an Optimised Fluctuations Score

Although the IQR_C_ does appear to distinguish fluctuators from non fluctuators, giving equal weighting to the BKS_IQR_ and DKS_IQR_ was arbitrary and empirical. Thus a general formula

$$IQR_{C}=s\;\times \;BKS_{IQR}+t\;\times \;DKS_{IQR}$$

was produced where *s* and *t* were independent weightings applied to BKS_IQR_ and DKS_IQR_. A family of IQR_C_ were produced by independently and serially varying *s* and *t* from 0.1 to 5.0 in steps of 0.1 when s or t<2 and otherwise in steps of 0.5. Each IQR_C_ was used to produce a p value by comparing the early PD and those on the DBS waiting list (in Fig 2B above). The p values varied from 0.6 to <0 0001, with the lowest being provided by a weighting of 1.0 for both *s* and *t*: i.e. the original IQR_C_.

Inspection of Fig 1 shows that the IQR_C_ is never less than 10 and that the IQR_C_ can become very large (the x axis was clipped at 100 with data points > 100 shown at that point). Thus the IQR_C_ was expressed as a logarithm with the offset of 10 removed to improve the visual effect in a plot. Thus a formula for Fluctuation Score (FS): FS = log^1.1^(IQR_C)_-25 (note that 25 is ~Log(10.8)), was subsequently used. Accordingly, the FS corresponding to the FT, as defined in the previous section, is 7.7.

### The change in FS following DBS

The FS was used to examine the changes in fluctuation that followed DBS surgery. The changes in clinical scales and in the PKG measures are shown in Table 1 and in Fig 3. The median BKS and median DKS represent scores from the period between 0900–1800 from all recording days. There was a statistical improvement in dyskinesia as measured by both clinical scales and PKG. There was a non-significant trend for both median BKS and UPDRS III to improve. There was also a significant improvement in the PKG’s Fluctuation Score. According to the FS, 7 of the 15 patients became non-fluctuators after DBS. According to the AIMS, most patients did not have dyskinesia after DBS (max AIMS = 3). There was a broad trend for the greatest improvement in FS to be in those with the highest FS before surgery (r^2^ = 0.57, data not shown). Six subjects with low FS score prior to surgery had only modest change in their clinical scores and in the case of two of these subjects, ~ 40% of the improvement in the UPDRS III was due to tremor, which is not captured in the FS.

**Table 1:** Scores from ratings scales and PKG before and after insertion of Deep Brain Stimulators.

	UPDRS III		AIMS		Median BKS		Median DKS		FS	
	before	after	before	after	before	after	before	after	before	after
No	15	14	15	14	15	15	15	15	15	15
Median	25	11.5	4	0	18.2	20.2	13.96	2.3	13.1	8.2
IQR	20	14	8	0.25	8.1	5.9	21.84	7.9	9.3	6.0
P value*	NS		0.01		NS		0.01		0.01	

* Mann Whitney

Conceptually, the state of motor fluctuations and dyskinesia in a cohort of patients who have received DBS could be taken as state in which these motor symptoms are under satisfactory control. If this was so, then it might be expected that DBS candidates whose FS score was in this range would see little change in motor fluctuations and dyskinesia following surgery because their state before and after surgery will be similar. If the FS is a measure of the extent of motor fluctuations and dyskinesia, then the 75^th^ percentile of FS (12.8) from the post-DBS patients is a reasonable upper range for an acceptable post DBS score and the FT (7.7) is a lower limit marking the loss of fluctuations. Using this reasoning, the range for control of motor symptoms (RCMS) by DBS would be a FS between 7.7 and 12.8.

## The natural history of the FS

An FS should also map the transition from non-fluctuator to fluctuator and this was examined by comparing the FS of subjects at various stages of PD (Fig 4A): these subjects are also shown in previous figures. Also, the FS of 38 subjects aged between 45 and 85 and without PD were plotted. There is a trend for the FS to progressively increase from early disease to late disease, even though the FS remained below the FT (FS = 7.7). The FS of early fluctuators had moved above the FT but for the most, remained within the RCMS. The median FS of established fluctuators was above the 75^th^ percentile of the post-surgery group. The FS of control subjects spanned, almost exactly, the range between the cut-off score and the 75^th^ percentile post-surgery.

The natural history of FS was further examined in 177 subjects (Fig 4B) selected retrospectively from all subjects who had worn the PKG logger for more than 5 days, whose time from symptom onset was known and who had also been assessed using AIMS. As this is a population attending a movement disorder clinic, it is biased toward fluctuating disease and younger patients. Their FS score was plotted against duration since first symptoms. In this cohort, FS scores within or above the RCMS became frequent after 3 years. On the other hand, some subjects maintained FS scores below the FT or else were in the low RCMS suggesting that they were less prone to develop fluctuations or were poorly responsive to dopaminergic stimulation. This cohort was then subdivided according to their AIMS (Table 2). The median FS of cases with an AIMS of 1 or 0 was 8.0 and in 65% of these cases FS were below the FT: the higher FS were mostly due to variation in the bradykinesia score. There was no statistical difference in the FS of subjects with AIMS between 2–5 and 6–9 so these were pooled and were statistical different to those with higher (>10) or lower AIMS. Broadly, subjects with AIMS of 2 or less, between 2–9 and greater than 10 approximated the FS below the FT, within the RCMS and above the RCMS (respectively).

**Table 2:** Relationship between AIMS and Fluctuation Score.

	AIMS				
	>2	2–5	6–9	2–9	>10
No. of subjects	96	25	16	41	34
25% Percentile of FS	6.8	8.8	8.1	8.5	12.2
Median of FS	8.0	9.8	9.4	9.7	14.6
75% Percentile of FS	9.7	14.7	12.6	14.3	20.3
Mann Whitney		●---P = 0.6---●		●--<0.0001--●	
	●-------------------P<0.005-------------------●				

## Discussion

When fluctuations in PD are more severe, variation in dyskinesia dominates the clinical picture. Similarly, variation in dyskinesia scores dominates the FS score when the IQR_C_ is large (Fig 1) and indeed variation in the DKS alone provides good discrimination between fluctuators and non-fluctuators. A combined BKS_IQR_ and DKS_IQR_ may provide better discrimination than the DKS_IQR_ alone because of the way “wearing-off” emerges. As the therapeutic benefit from levodopa shortens, under-treatment emerges prior to the next dose, reflected as “wearing-off” and as variation in the bradykinesia score. Another consideration is that after the transition from non-PD state to PD (Fig 4) there is loss of variation in the BKS and DKS, but particularly the BKS. Therapy aimed at overcoming bradykinesia, will also restore “normal” variation in the BKS. Indeed the art of PD therapy is to find the line between minimising bradykinesia without inducing dyskinesia: i.e. minimising fluctuations in both scores. For whatever the reason however, it is apparent that the FS that detects emergent fluctuations requires a significant contribution from variation in both BKS and DKS as shown in Fig 1B.

The concept behind the PKG’s algorithms for bradykinesia and dyskinesia is that kinetic characteristics of the movements of non-PD subjects will have varying degrees of similarity with the bradykinesia or dyskinesia of PD and are scored according to that similarity[9]. Thus both non-PD subjects and PD patients will have a distribution of movements; and bradykinesia or dyskinesia scores reflect the degree to which these movements deviate from the distribution of the normal population. While a measurement, such as the BKS or the UPDRS III, will provide a snapshot of a person’s kinetic state at a particular time there is considerable variability in movement throughout the day. All the BKS and DKS collected over a recording period represent a distribution or population of movements made by the individual and the median BKS and DKS are measures of the central tendency of this population. On the other hand the FS is a measure of the variability of this population: in other words its tendency to fluctuate. Diaries are an attempt to provide measures that are not locked to a single point in time. Apart from the well documented shortcomings of diaries[10] they do not have the dynamic range or frequency that the FS has. We suggest therefore that FS has the potential to become an important tool in assessing fluctuations. It is interesting therefore that the transition to untreated PD (Fig 4A) results in loss of this variation as measured by the FS and also that DBS appears to bring the level of variation in dyskinesia into a range that approximates “normal” fluctuation (Fig 3). The idea of a range for control of motor symptoms (RCMS) by DBS or other advanced therapies is that there is some “best” level of function, beyond which further improvement is both uncommon and unlikely. If this is the case then subjects whose FS is within the RCMS prior to DBS would not expect improvement in FS, although DBS might maintain them at this level without the excess fluctuations developing. In this study the RCMS has been defined from a small number of people receiving DBS and a large cohort would be required to definitively establish this as the therapeutic target for DBS. However it is of interest that the RCMS found with this small cohort lies close to the FT and also approximates the interquartile range of subjects who do not have PD. DBS is effective in abolishing dyskinesia and improving “wearing-off” so it is likely that the lower range of the RCMS would be close to the FT, implying absence of fluctuations.

The data presented here points to the possibility that a non fluctuator has an FS below a discreet threshold (the FT) and a controlled fluctuator has an FS in the bottom half of the RCMS. Another interpretation might be that there is a “normal” range defined by the interquartile range of non-PD subjects or the bottom half of the RCMS: below this is relative under treatment whereas above this also requires attention for excess movements. Fig 4B suggest that patients either persist with FS in, or just below the lower half of the RCMS for many years of disease or else transition to scores well above the RCMS. This data is drawn from a specialist clinic and may under represent the patients with low FS who are unresponsive to levodopa. We conclude from this data (Fig 4C) that the FS falls progressively as PD develops and that with treatment (and time) the FS score progressively climbs toward the FT and into the RCMS where patients will follow one of two paths. They will either become frank fluctuators or will remain in or just below the lower half of the RCMS. This second path suggests a benign course but it may also include patients that are poorly responsive to dopaminergic treatment. Recognising those who are destined for the fluctuation pathway of Fig 4C would lead to timely and effective intervention with DBS.

In Summary, we propose that an FS derived from variation in the BKS and DKS of the PKG has potential as a tool for choosing and optimising therapies for patients with PD. Further studies in large cohorts of patients are required to provide a more definite description of the FT and RCMS as described here. Future studies may also better aid in confirming the hypothesis behind Fig 4C.
